# Differential dosimetric benefit of proton beam therapy over intensity modulated radiotherapy for a variety of targets in patients with intracranial germ cell tumors

**DOI:** 10.1186/s13014-015-0441-5

**Published:** 2015-06-26

**Authors:** Jeonghoon Park, Younghee Park, Sung Uk Lee, Taeyoon Kim, Yun-Kyung Choi, Joo-Young Kim

**Affiliations:** Center for Proton Therapy, Goyang-si, Republic of Korea; Department of Radiation Oncology, Soonchunhyang University Hospital, Seoul, Republic of Korea; Center for Pediatric Cancer, National Cancer Center, Goyang-si, Republic of Korea

**Keywords:** Intracranial germ cell tumors, Proton beam therapy, Pencil beam scanning, IMRT

## Abstract

**Background:**

We performed dosimetric comparisons between proton beam therapy and intensity modulated radiotherapy (IMRT) of intracranial germ cell tumors (ICGCTs) arising in various locations of the brain.

**Materials:**

IMRT, passively scattered proton therapy (PSPT), and spot scanning proton therapy (SSPT) plans were performed for four different target volumes: the whole ventricle (WV), pineal gland (PG), suprasellar (SS), and basal ganglia (BG). Five consecutive clinical cases were selected from the patients treated between 2011 and 2014 for each target volume. Total 20 cases from the 17 patients were included in the analyses with three overlap cases which were used in plan comparison both for the whole ventricle and boost targets. The conformity index, homogeneity index, gradient index, plan quality index (PQI), and doses applied to the normal substructures of the brain were calculated for each treatment plan.

**Results:**

The PQI was significantly superior for PSPT and SSPT than IMRT for ICGCTs in all locations (median; WV: 2.89 and 2.37 vs 4.06, PG: 3.38 and 2.70 vs 4.39, SS: 3.92 and 2.49 vs 4.46, BG: 3.01 and 2.49 vs 4.45). PSPT and SSPT significantly reduced the mean dose, and the 10 and 15 Gy dose volumes applied to the normal brain compared with IMRT (*p* ≤ 0.05). PSPT and SSPT saved significantly greater volumes of the temporal lobes and hippocampi (*p* < 0.05) in the SS and PG targets than IMRT. For tumors arising in the BG, PSPT and SSPT also saved greater volumes of the contralateral temporal lobes.

**Conclusions:**

PSPT and SSPT provide superior target volume coverage and saved more normal tissue compared with IMRT for ICGCTs in various locations. Future studies should assess whether the extent of normal tissue saved has clinical benefits in children with ICGCTs.

**Electronic supplementary material:**

The online version of this article (doi:10.1186/s13014-015-0441-5) contains supplementary material, which is available to authorized users.

## Background

Intracranial germ cell tumors (ICGCTs) include tumors of various histologic subtypes that arise from the primordial germ cell of developing embryos. They are rare in western countries, accounting for 0.5–3 % of childhood brain tumors. However, their incidence is higher in Taiwan, Japan, and Korea, where ICGCTs account for 11–14 % of all brain tumors in children aged < 19 years [1–3].

Although chemotherapy is widely used with the aim of reducing radiotherapy volume, the focal radiation field in combination with chemotherapy before radiotherapy actually increase the risk of local and regional recurrence [4]. The current consensus for treating localized ICGCTs involves delivery of radiotherapy to a sufficient target of the entire ventricular space, irrespective of the use of chemotherapy [4–8]. However, there are concerns about the potential adverse effects of applying radiotherapy to a large volume on long-term neurocognitive effects and quality of life. Although the general cognitive abilities of most children were intact after treatment even if they received craniospinal irradiation [9], another study showed that there were significant declines over time in working memory and processing speed [10]. In a Japanese study [11], among 34 patients of University of Niigata with intracranial germinoma who were older than 19 at the time of follow-up, only 18 (52 %) patients had jobs in ordinary (unsheltered) work places and 8 (24 %) patients were unemployed at the time of follow-up.

Because disease-related morbidities and treatment-related complications are the major contributors to the long-term decline in brain function, it is important to minimize the relative dose and volume of the normal tissue to those of target tissue without compromising the treatment outcome. Proton beam therapy (PBT) is an important radiotherapeutic modality that may improve the neurocognitive outcomes of children with brain tumors because of its dosimetric advantage compared with other modalities [12–14]. PBT can deliver a lower radiation dose to normal tissues proximal and distal to tumors owing to unique depth dose distribution.

A previous study that examined the relationship between radiation dose and cognitive effects suggested that applying low doses to subvolumes of the brain and the reduced mean dose of proton beam therapy would translate into long-term clinical advantages in several common brain tumors [15]. It was also demonstrated that increasing the radiotherapy dose applied to the hippocampus and temporal lobes is associated with a greater decline in neurocognitive skills following cranial irradiation [16]. In this study, we evaluated whether PBT offers dosimetric benefits over intensity modulated radiotherapy (IMRT) for the treatment of ICGCTs arising in various locations of the brain with 20 ICGCT cases selected from the single institutional database.

## Methods

### Patients and treatment targets

Between April 2007 and February 2014, a total of 50 patients with ICGCTs were treated at the National Cancer Center, Korea. Passively scattered proton beam, i.e. double scattering technique, was used to treat 36 patients with ICGCTs. Seventeen consecutive patients with localized ICGCTs were included in this study. The two patients with pineal tumor and one with suprasellar tumor were given WVI before boosting the primary site. These three cases were used for comparing plans of both the WVI and primary site irradiation.

All patients received four cycles of chemotherapy before radiotherapy according to the treatment scheme proposed by the Korean Society for Pediatric Neuro-Oncology. Regarding radiotherapy, WVI of 19.8 Gy was given initially, and the field was reduced to the primary site and was given 30.6–39.6 Gy, depending on the tumor’s response to chemotherapy. All patients with non-germinomatous germ cell tumors (NGGCT) received craniospinal irradiation (CSI) of 36 Gy and the primary site was irradiated with 55.8–59.4 Gy depending on the tumor’s response to chemotherapy.

### Treatment planning

The treatment plans were compared to investigate the effectiveness of PBT. Brain CT images were obtained with slice thickness of 2.5 mm using a Q-fix patient support (Q-Fix, Avondale, PA, USA). For all patients, the treatment targets and normal intracranial structures, including normal brain, brainstem, temporal lobe, hippocampus, cochlea, lens, optic nerve, optic chiasm, hypothalamus, and pituitary gland, were contoured by single radiation oncologist using the Eclipse® treatment planning system (TPS) v10.0.2 (Varian Medical Systems, Palo Alto, CA, USA). For target contouring, MRI was performed at the same time as planning CT (after completing chemotherapy) and at the time of diagnosis (before starting chemotherapy), and data were anatomically registered with the planning CT. The WV gross tumor volume (GTV) included the lateral, third, and fourth ventricles, and the prepontine cistern. The WV clinical target volume (CTV) was defined as the WV GTV + 0.7 cm and the WV PTV was defined as the CTV + 0.3 cm. The tumor bed GTV was contoured based on the volume measured before chemotherapy for the primary site boost. Margins of 1–1.5 cm were added to the GTV to define the CTV, taking into account the anatomic borders of the adjacent tissues and the changes in tumor bed following surgery or chemotherapy. The PTV for the primary site boost was defined as the CTV + 0.3 cm.

The IMRT and PBT plans were prepared for all treatment targets using the same CT images and structure sets. To facilitate comparisons, the same 30.6 Gy dose was prescribed to all PTV volumes and the treatment plans were normalized to 100 % of the dose across 95 % of the PTV volume. The IMRT plans were optimized using seven coplanar beams of 6 MV that were equally spaced around the head. The brainstem, cochlea, eye, lens, optic nerve, optic chiasm, temporal lobe, pituitary gland, hippocampus were included in the plan optimization as constraint structures. The dose distribution was calculated using the analytic anisotropic algorithm by the Eclipse TPS with a Varian 21EX linear accelerator.

Two types of PBT were used, passively scattered proton therapy (PSPT) and spot scanning proton therapy (SSPT). PSPT applies the spread-out Bragg peak to fit a pristine Bragg peak to a longitudinal depth of tumor using a range modulator. Blocks and compensators are also used to shape the lateral beam margin and the distal tumor shape, respectively. In PSPT planning, the block margin was set to 0.7–0.8 cm and the proximal/distal margins were set to 0.2 cm from the PTV. Compensator border smoothing and smearing were set to 0.8 and 0.3 cm, respectively. SSPT uses a narrow pencil beam modulated only by transverse, longitudinal scanning magnets in the nozzle. In our institution, the nominal beam spot size (σ) ranges from 5.8 mm at 32 g/cm^2^ to 11 mm at 7.5 g/cm^2^ using an Ion Beam Application universal nozzle (Ion Beam Applications, Louvain-la-Neuve, Belgium). The SSPT plans were developed using the Eclipse TPS with an inverse planning feature in the single field optimization mode, which optimizes dose spots in a similar way to PSPT. In SSPT planning, a spot spacing of 0.7 cm and a lateral margin of 0.7 cm were used. The proton beam plans were calculated with the proton convolution superposition algorithm of the Eclipse TPS. The PSPT and SSPT plans were created using three beams while maintaining the beam directions relative to each other. For BG planning, posterior–anterior, anterior–superior–oblique, and lateral beams were used through the side on which the tumor was located. Posterior–anterior and both lateral beams were used in WV plans, while other plans were prepared using superior oblique beams applied at equal beam angles along the midline to minimize the dose applied to the temporal lobes.

### Dosimetric comparison and statistical analysis

For all treatment plans, cumulative dose–volume histograms (DVH) were exported for all structures with relative dose bins of 0.1 %. For each PTV, we calculated the minimum, maximum, and mean dose, the minimum doses applied to 95 and 5 % of the PTV volume (D95 % and D5 %, respectively), the dose volume receiving more than 100 or 50 % the prescribed dose (V100 % [prescribed isodose volume] and V50 %, respectively), and the PTV volume covered by the prescribed dose. Using these data, we calculated the conformity index (CI), homogeneity index (HI), gradient index (GI) and the plan quality index (PQI) by combining CI, HI, and GI. The definitions of CI, HI, GI and PQI are presented in the Additional file 1, available online. The ideal plan gives those indices of 1.0. PQI is similar to the unified dosimetry index, an efficient tool for ranking treatment plans [17]. PQI has an ideal value of 1.0 if the plan’s target volume conforms exactly to the prescribed dose. Using the PQI, the diversities of CI, HI, and GI in different plans can be transferred into a single comparison index.

Of the normal structures contoured during planning, the normal brain, both temporal lobes, both hippocampi, and the pituitary gland were selected for analysis. The mean doses applied to these organs were analyzed. The volumes of normal brain that received absolute doses of > 10 Gy or > 15 Gy were calculated. In WV irradiation, both hippocampi were excluded from the analysis because of their close proximities to the target volume. For BG tumors, the temporal lobes and hippocampi were termed ipsilateral or contralateral depending on the tumor’s location. The dosimetric outcomes of IMRT, PSPT, and SSPT were compared using the Wilcoxon signed-rank test because the sample size of our data is small (5 cases in each set). The outcomes were considered statistically significant at *p*-values of < 0.05. All statistical analyses were performed using SPSS software version 18.0 (SPSS Inc., Chicago, IL, USA). Also, the dose distributions are shown for a representative patient with a WV irradiation and primary boost irradiation planned with IMRT and PSPT technique, respectively. Then, the sum of the WV dose and the primary site boost were compared each other.

## Results

### Characteristics of the patients and the treatment targets

The patients and target volume characteristics are summarized in Table 1. Of 17 patients included in this study, 10 had pure germinomas and 7 had NGGCTs. None of the patients had disseminated disease or bifocal tumors. The mean ± standard deviation (range) WV PTV volume was 415.1 ± 63.6 cm^3^ (330.6–504.7 cm^3^), and varied according to the patient’s head size and the presence of residual hydrocephalus at the time of PBT. The PTV volumes were 103.9 ± 62.5 cm^3^ (33.6–169.0 cm^3^), 61.3 ± 20.2 cm^3^ (44.7–87.9 cm^3^), and 124.2 ± 21.3 cm^3^ (87.4–142.1 cm^3^), for PG, SS and BG, respectively. The BG tumors were located on the left side in three patients and the right side in two patients. The median follow-up was 18.7 months (range, 5.8–32.9 months) for 14 patients, and none of the 14 patients experienced tumor recurrence after PBT. The follow-up data were not available for the other 3 patients.

**Table 1:** Patients and PTV volume characteristics

Characteristics			Value
Sex			
	Male		13
	Female		4
Histology			
	Germinoma		10
	Mixed germ cell tumor		7
PTV location and volume			
	Whole Ventricle (cases)		5
		Mean ± SD (cc)	415.1 ± 63.6
		Range (cc)	330.6–504.7
	Pineal Gland (cases)		5
		Mean ± SD (cc)	103.9 ± 62.5
		Range (cc)	33.6–169.0
	Suprasellar (cases)		5
		Mean ± SD (cc)	61.3 ± 20.2
		Range (cc)	44.7–87.9
	Basal Ganglia (cases)		5
		Left : Right	3 : 2
		Mean ± SD (cc)	124.2 ± 21.3
		Range (cc)	87.4–142.1

### Comparison of PTV Coverage

The results of the dosimetric comparison and statistical analysis of PTV coverage are shown in Table 2. Regarding the conformity of the PTV, the CI was better for SSPT than both IMRT and PSPT (both *p < 0.05*), and was lower for PSPT than IMRT. The GI was comparable in PSPT and SSPT, but was better for both of these modalities than IMRT (both *p* = 0.043). The HI was similar for all three modalities. The PQI decreased significantly in the order of IMRT > PSPT > SSPT in all target locations (median, WV: 4.06, 2.89 and 2.37; PG: 4.39, 3.38 and 2.70; SS: 4.46, 3.92 and 2.49; BG: 4.45, 3.01 and 2.49). Based on the PQI—a single dosimetric index that combines CI, GI, and HI—the PSPT and SSPT plans seem to be superior to the IMRT plan and the SSPT plan is superior to the PSPT plan in terms of PTV coverage.

**Table 2:** Dosimetric comparison and statistical analysis of PTV coverage

Item	PTV location	Median (range, min–max)			p value^*^		
		IMRT	PSPT	SSPT	IMRT vs PSPT	IMRT vs SSPT	PSPT vs SSPT
PTV CI	WV	0.85 (0.72–0.88)	0.70 (0.69–0.71)	0.91 (0.89–0.92)	<0.05	<0.05	<0.05
	PG	0.92 (0.87–0.92)	0.83 (0.62–0.87)	0.94 (0.90–0.90)	<0.05	<0.05	<0.05
	SS	0.89 (0.88–0.92)	0.74 (0.72–0.83)	0.92 (0.90–0.94)	<0.05	<0.05	<0.05
	BG	0.88 (0.86–0.91)	0.80 (0.74–0.82)	0.94 (0.93–0.95)	<0.05	<0.05	<0.05
PTV GI	WV	3.30 (3.08–3.75)	1.90 (1.85–2.04)	2.12 (1.99–2.19)	<0.05	<0.05	<0.05
	PG	3.89 (3.47–4.62)	2.90 (2.50–3.25)	2.50 (2.36–3.34)	<0.05	<0.05	NS
	SS	3.82 (3.71–4.14)	2.78 (2.64–3.27)	2.59 (2.48–2.87)	<0.05	<0.05	NS
	BG	3.77 (3.46–4.23)	2.35 (2.18–2.57)	2.30 (2.23–2.60)	<0.05	<0.05	NS
PTV HI	WV	0.97 (0.96–0.98)	0.95 (0.94–0.95)	0.98 (0.98–0.98)	<0.05	<0.05	<0.05
	PG	0.96 (0.95–0.97)	0.97 (0.96–0.98)	0.98 (0.97–0.98)	NS	<0.05	NS
	SS	0.96 (0.95–0.97)	0.97 (0.96–0.98)	0.97 (0.95–0.98)	NS	NS	NS
	BG	0.97 (0.97–0.98)	0.97 (0.97–0.98)	0.97 (0.97–0.98)	NS	NS	<0.05
PTV PQI	WV	4.06 (3.82–4.73)	2.89 (2.75–3.11)	2.37 (2.23–2.46)	0.043	0.043	0.043
	PG	4.39 (3.92–5.55)	3.38 (3.12–5.47)	2.70 (2.54–3.83)	0.043	0.043	0.043
	SS	4.46 (4.26–4.88)	3.92 (3.62–4.05)	2.95 (2.88–3.12)	0.043	0.043	0.043
	BG	4.45 (4.00–4.93)	3.01 (2.81–3.59)	2.49 (2.44–2.86)	0.043	0.043	0.043

*WV* whole ventricle, *PG* pineal gland, *SS* suprasellar, *BG* basal ganglia, *PTV* planning target volume, *CI* conformity index, *GI* gradient index, *HI* homogeneity index, *PQI* plan quality index, *IMRT* intensity-modulated radiation therapy, *PSPT* passively scattered proton therapy, *SSPT* spot scanning proton therapy, *NS* not significant

^*^ *p* -value by Wilcoxon signed rank test

### Normal organ sparing

**Fig. 1:**
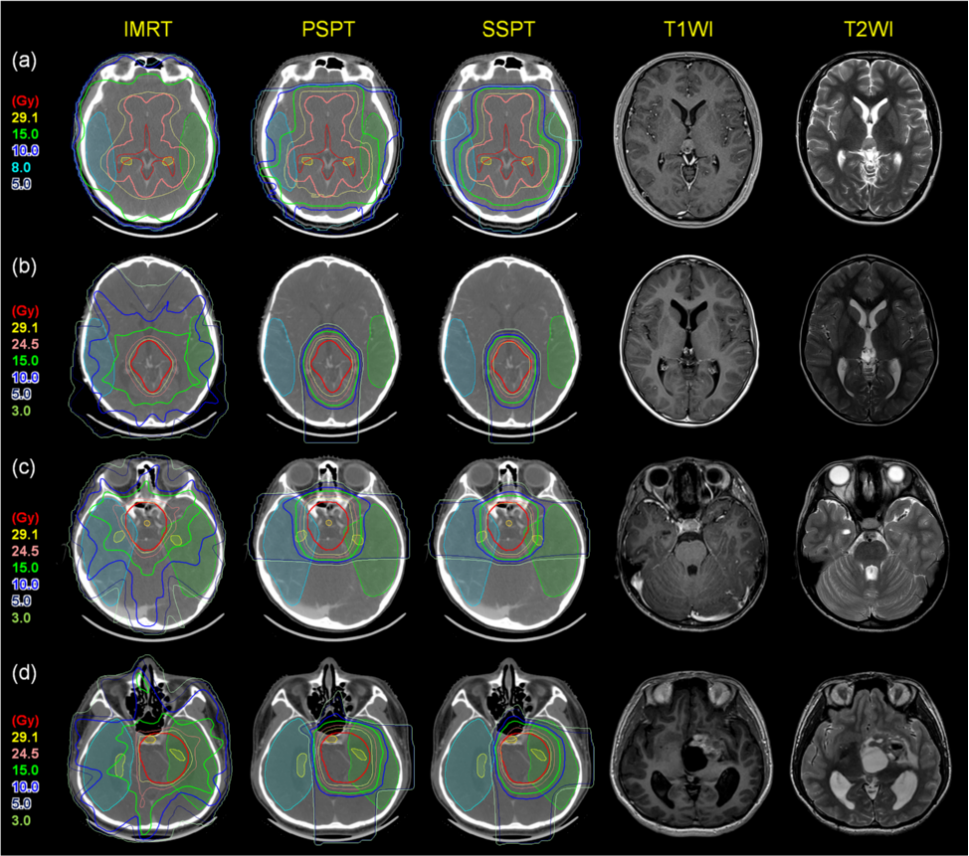
Dose distributions of WV irradiation and PG, SS and BG tumor beds with their pre-chemotherapy MRI images. Thick pink line is WV PTV, and thick red lines are tumor beds PTV. Prescribed dose are 30.6 Gy at 95 % volume of PTV. (**a**) WV (**b**) PG (**c**) SS (**d**) BG

**Fig. 2:**
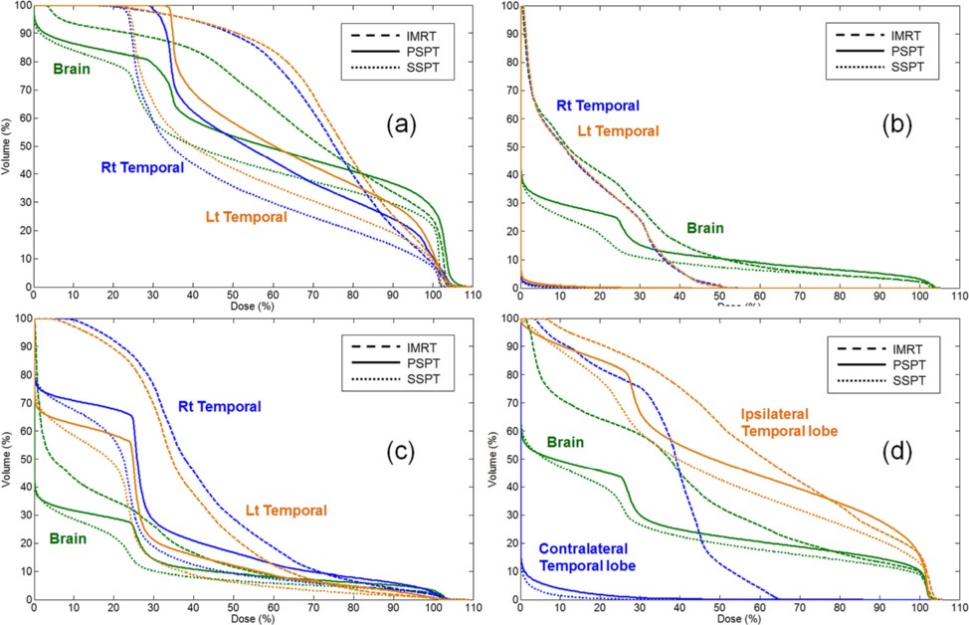
Dose volume histogram (DVH) of normal brain and both temporal lobes in the WV, PG, SS and BG treatment plans. (**a**) WV (**b**) PG (**c**) SS (**d**) BG

PSPT and SSPT were associated with significant reductions in the mean dose (median, WV: 19.8 Gy and 17.5 Gy vs 22.1 Gy; PG: 8.3 Gy and 7.0 Gy vs 10.5 Gy; SS: 4.4 Gy and 3.5 Gy vs 5.8 Gy; BG: 8.8 Gy and 7.7 Gy vs 12.4 Gy for PSPT and SSPT vs. IMRT, respectively), and the 10 Gy (median, WV: 77.9 and 62.7 % vs 88.5 %; PG: 25.4 and 21.1 % vs 47.8 %; SS: 12.7 and 9.4 % vs 23.0 %; BG: 29.1 and 25.2 % vs 56.5 %) and 15 Gy dose volumes (median, WV: 60.1 and 53.5 % vs 79.5 %; PG: 25.4 and 21.1 % vs 26.1 %; SS: 9.4 and 7.1 % vs 11.9 %; BG: 24.3 and 20.1 % vs 33.6 %) applied to the normal brain compared with IMRT for all four target volumes (all *p ≤* 0.05, Table 3). The dose distributions applied to the normal brain in each target location are shown in Fig. 1. Figure 2 shows the DVH for four treatment plans for target volumes in four different locations. PSPT and SSPT were associated with lower doses applied to the normal brain for all four target locations.

**Table 3:** Dosimetric comparison and statistical analysis of dose to normal brain and substructures

PTV location	Item	Median (range, min–max), Gy			p value^*^		
		IMRT	PSPT	SSPT	IMRT vs PSPT	IMRT vs SSPT	PSPT vs SSPT
WV	Brain - D_mean_	22.1 (21.2–23.3)	19.8 (18.4–21.3)	17.5 (15.9–18.7)	<0.05	<0.05	<0.05
	Brain - V10Gy (%)	88.5 (85.5–90.0)	77.9 (74.8–82.1)	62.7 (55.6–66.5)	<0.05	<0.05	<0.05
	Brain - V15Gy (%)	79.5 (75.9–83.6)	60.1 (54.0–64.8)	53.5 (45.5–57.0)	<0.05	<0.05	<0.05
	Rt. Temporal	22.5 (18.6–25.8)	22.5 (18.6–25.8)	19.6 (14.8–23.7)	<0.05	<0.05	<0.05
	Lt. Temporal	22.4 (18.9–27.2)	22.4 (18.9–27.2)	19.4 (15.4–25.6)	<0.05	<0.05	<0.05
	Rt. Hippocampus	31.3 (30.4–31.6)	31.3 (30.4–31.6)	31.1 (27.4–31.2)	<0.05	NS	<0.05
	Lt. Hippocampus	31.4 (31.2–31.5)	31.4 (31.2–31.5)	31.1 (28.7–31.2)	NS	<0.05	<0.05
	Pituitary Gland	31.8 (30.9–31.9)	31.8 (30.9–31.9)	31.0 (30.7–31.4)	NS	NS	NS
PG	Brain - D_mean_	10.5 (6.5–14)	8.3 (4.4–12.2)	7.0 (3.3–10.4)	<0.05	<0.05	<0.05
	Brain - V10Gy (%)	47.8 (24.1–63.9)	25.4 (13.9–42.3)	21.1 (10.2–35.8)	<0.05	<0.05	NS
	Brain - V15Gy (%)	26.1 (10.9–41.0)	20.5 (10.5–34.7)	16.4 (7.5–27.8)	<0.05	<0.05	<0.05
	Rt. Temporal	15.0 (4.8–21.2)	2.8 (0.0–10.8)	2.0 (0.0–8.8)	<0.05	<0.05	NS
	Lt. Temporal	14.7 (4.8–20.5)	2.9 (0.1–8.5)	3.0 (0.0–7.4)	<0.05	<0.05	NS
	Rt. Hippocampus	28.0 (10.9–31.2)	25.1 (6.4–31.5)	21.8 (4.8–30.9)	NS	<0.05	NS
	Lt. Hippocampus	25.8 (15.3–30.9)	23.0 (11.0–31.5)	22.3 (8.4–30.6)	NS	<0.05	<0.05
	Pituitary Gland	6.8 (0.7–23.5)	5.0 (0.0–29.3)	3.2 (0.0–27.7)	NS	NS	NS
SS	Brain - D_mean_	5.8 (4.9–11)	4.4 (3.6–7.9)	3.5 (2.9–6.7)	<0.05	<0.05	<0.05
	Brain - V10Gy (%)	23.0 (18.3–49.0)	12.7 (10.2–27.1)	9.4 (7.6–22.6)	<0.05	<0.05	<0.05
	Brain - V15Gy (%)	11.9 (9.8–25.9)	9.4 (7.6–21.8)	7.1 (5.6–17.2)	<0.05	<0.05	<0.05
	Rt. Temporal	12.9 (8.8–15.8)	6.6 (4.3–9.9)	5.1 (3.4–6.9)	<0.05	<0.05	<0.05
	Lt. Temporal	11.9 (9.1–15.4)	6.4 (3.0–9.5)	5.0 (2.4–6.7)	<0.05	<0.05	<0.05
	Rt. Hippocampus	15.9 (11.7–17.5)	10.1 (4.6–16.1)	7.2 (2.4–12.6)	<0.05	<0.05	<0.05
	Lt. Hippocampus	16.2 (12.7–17.5)	11.0 (6.2–15.3)	7.8 (3.7–13.5)	<0.05	<0.05	<0.05
	Pituitary Gland	31.4 (5.9–31.6)	31.2 (15–31.4)	31.4 (14.4–32.2)	NS	NS	NS
BG	Brain - D_mean_	12.4 (11.6–14.2)	8.8 (7.5–9.9)	7.7 (6.3–8.4)	<0.05	<0.05	<0.05
	Brain - V10Gy (%)	56.5 (52.9–66.5)	29.1 (26.5–34.1)	25.2 (22.5–28.9)	<0.05	<0.05	<0.05
	Brain - V15Gy (%)	33.6 (28.8–39.9)	24.3 (21.7–28.0)	20.1 (17.7–23.2)	<0.05	<0.05	<0.05
	Ipsilat. Temp.	19.0 (13.2–22.6)	17.0 (13.8–23.1)	15.1 (10.4–20.3)	NS	<0.05	<0.05
	Contralat. Temp.	10.3 (6.5–12.1)	0.0 (0.0–0.4)	0.0 (0.0–0.2)	<0.05	<0.05	NS
	Ipsilat. Hippoc.	29.1 (24.3–31)	30.4 (27.1–31.2)	29.7 (24.6–31.1)	NS	NS	NS
	Contralat. Hippoc.	15.6 (9.2–16.7)	0.2 (0.0–0.7)	0.1 (0.0–0.6)	<0.05	<0.05	NS
	Pituitary Gland	12.5 (1.4–29.9)	22.4 (0.0–30)	11.5 (0.0–29.3)	NS	NS	NS

*WV* whole ventricle, *PG* pineal gland, *SS* suprasellar, *BG* basal ganglia, *PTV* planning target volume, *IMRT* intensity-modulated radiation therapy, *PSPT* passively scattered proton therapy, *SSPT* spot scanning proton therapy, *NS* not significant

^*^ *p* -value by Wilcoxon’s signed rank test

The doses applied to the temporal lobe and hippocampus according to the target locations are compared among the treatment plans in Table 3. The DVH for each plan is shown in Fig. 2. PSPT and SSPT saved significantly greater volumes of the temporal lobes compared with IMRT in all targets. PSPT and SSPT had the greatest benefit in terms of saving the contralateral temporal lobe for BG targets with median doses of 10.3 Gy (range, 6.5–12.1 Gy) for IMRT, 0.0 Gy (range, 0.0–0.4 Gy) for PSPT, and 0.0 Gy (range, 0.0–0.2 Gy) for SSPT. SSPT also saved greater volumes of the temporal lobe, even on the ipsilateral side, than PSPT (median, 15.1 Gy vs 17.0 Gy; *p ≤* 0.05). In the hippocampus, PSPT and SSPT were similar to IMRT for tumors located in the WV and PG, but had significant benefits over IMRT for SS and BG targets. For SS targets, the dose reduction was greater for SSPT than IMRT and PSPT. For BG targets, the dose reduction in the hippocampus was similar to that in the temporal lobe, suggesting greater benefits of PSPT and SSPT in terms of saving the contralateral hippocampus. The median doses applied to the hippocampus were 15.6 Gy (range, 9.2–16.7 Gy) for IMRT, 0.2 Gy (range, 0.0–0.7 Gy) for PSPT and 0.1 Gy (range, 0.0–0.6 Gy) for SSPT. The dose distribution of the BG plans shown in Fig. 1d confirms these results.

The median doses applied to the pituitary gland are also compared in Table 3. Unlike other organs, there were no significant benefits of PSPT and SSPT in terms of sparing the pituitary gland. For the WV and SS targets, the pituitary gland was included in the PTV because the target volumes included the third ventricle and the prepontine cistern. Therefore, the pituitary gland could not be spared with any treatment plan. Although SSPT could reduce the dose applied to the pituitary gland for BG targets, IMRT offered a similar level of protection to SSPT (median, 12.5 Gy vs 11.5 Gy; *p* > 0.05).

### Dose distributions in a representative case

**Fig. 3:**
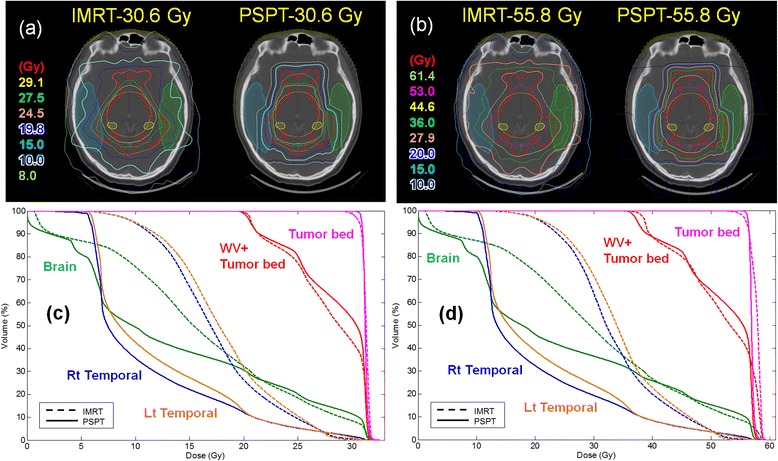
Comparison of dose distribution and DVH of combined treatment plans for PG tumor bed with IMRT and PSPT. (**a**, **c**) WV 19.8 Gy + tumor bed 10.8 Gy (**b**, **d**) WV 36 Gy + tumor bed 19.8 Gy

Figure 3 shows the dose distributions of a representative case with a combined plan (WV and a tumor bed boost). The IMRT and PSPT plans were compared using two different prescriptions: (1) WV dose of 19.8 Gy and a tumor bed boost of 10.8 Gy (germinoma) and (2) WV dose of 36 Gy and a tumor bed boost of up to 55.8 Gy (mixed germ cell tumor). PSPT saved a significant proportion of the normal organs with isodose volumes of 20–70 % in combined plans comprising a WV or WB dose and a tumor boost. In the 55.8 Gy plan, PSPT had an apparent dose benefit over IMRT. As shown in Fig. 3c, the 20 Gy line includes the entire brain with IMRT, but only the 10 Gy line is marginal with PSPT.

## Discussion

The radiotherapy principle for ICGCTs is to cover the cerebrospinal fluid pathway in the brain sufficiently so that the microscopic tumor cells located around the ventricular system are fully irradiated. However, the temporal lobes and the hippocampi which are important in learning and memory receive a considerable amount of radiation dose while performing WV and boost irradiation to the tumors which are typically located along the midline of the brain. So, they need to be protected from irradiation as much as possible.

In a previous study, MacDonald et al. compared IMRT, PSPT, and SSPT plans for treating pediatric ICGCTs, and described the clinical outcomes of PSPT [18]. They reported a superior dose distribution of SSPT over the other planning methods, similar to our results. For tumors with relatively small volumes, the normal brain volume that receives low to intermediate radiation dose is remarkably low in proton beam therapy compared with the IMRT or 3D CRT. For WV targets, the dosimetric benefit of PBT in terms of D_mean_, V_10 Gy_ and V_15 Gy_ may seem less prominent because of the relatively small volume of normal tissue outside WV in the brain. However, for large tumors arising in other body sites such as large retroperitoneal sarcoma or large chest wall tumors, sparing effect of normal tissue of PBT in terms of absolute volume of the normal tissue would be much larger than the effect that can be observed in treatment of brain tumors.

We found that PSPT and SSPT provided better PTV coverage and achieved greater dose reductions for organs at risk, especially the normal brain and temporal lobes. Regarding WV irradiation with a dose of 30.6 Gy, the 10 Gy and 15 Gy dose volumes were significantly lower for both PBT techniques than for IMRT. When we compared the mean doses applied to the normal brain and temporal lobes, we found that the dose was 10 % lower for PSPT than for IMRT, and was 10 % lower for SSPT than for PSPT. The superiority of SSPT over PSPT is due to a lower proximal dose applied to the normal brain as a result of a lack of range modulation. The range modulation and use of compensator is associated with an increased normal tissue irradiation and potential side effects such as alopecia [19, 20]. Systematic studies are needed to examine whether this reduction in the radiation dose translates into clinically detectable improvements in neurocognitive function. Previous studies showed that when irradiating the WV, IMRT successfully reduced the volume of the hemispheric brain volume exposed to low doses, but this was achieved at the expense of delivering an unnecessary radiation dose to the periphery of the body though increased leakage radiation [21]. Accordingly, SSPT appears to be an excellent tool with improved conformity and reduced neutron contamination compared with other radiotherapy modalities [22]. However, it should be always born in mind that SSPT plans are more sensitive to treatment uncertainties such as proton range uncertainty, dose calculation uncertainty, and inter-fractional and inter-field motions compared to PSPT. So, robust plan optimization should be considered in SSPT plans to minimize adverse effects [23–25].

In addition to the WV target, PBT had dosimetric benefits for the other targets. The advantage of PBT in treating SS tumors can be compared to a study of craniopharyngiomas or optic gliomas arising in similar locations where reductions in low to intermediate doses improved the intelligent quotient and academic reading scores in a dose–cognitive model [15]. Although ICGCTs located in the BG are infrequent, about 16 and 13 % of germinomas and NGGCTs, respectively, were located in the BG in patients registered between 2005 and 2011 in the Korean ICGCT registry ((personal communication, Chang-Ok Suh, the Korean Society for Pediatric Neuro-Oncology)). Similar incidences were reported in Taiwan, Hong Kong, and Japan [11, 26, 27]. Patients with tumors located in the BG are at increased risk of reduced neurocognitive function and worse performance status [11, 28, 29]. The reasons for these outcomes are complex, but the tumor location might contribute to poor physical performance because tumors in the BG can affect processing speed and might be diagnosed much later than ICGCTs in other locations. A radiation field with sufficient margins around the gross tumor is needed because of the diffuse infiltrative tumor growth in most patients. PBT significantly reduced the dose applied to the contralateral temporal lobe and the hippocampus because of the Bragg peak phenomenon, even though the beams pass through the ipsilateral side. In combination with WV irradiation or CSI, the dose applied to the contralateral temporal lobe and hippocampus was reduced by 10–20 Gy for germinomas and NGGCTs.

We used the PQI to help compare the treatment plans between different modalities. The CI and GI, two commonly used dosimetric indexes, could not precisely depict the dose distributions of each modality. PSPT was associated with the lowest CI because of the proximal high-dose region around the PTV arising from the uniform spread-out Bragg peak. The CI was significantly better for IMRT and SSPT than PSPT because of the high modulation of incident radiation. However, the GI was inferior for IMRT because of the use of multiple beams and the high exit dose after the PTV. By contrast, the GI for PSPT was similar to that of SSPT because of the use of blocks and the effects of the Bragg peak. Finally, the HI was similar for all plans. Considering these factors, it is difficult to compare each plan and determine which plan offers the best target coverage. This highlights the need for a single dosimetric index to compare different treatment plans, like the PQI used in the present study. Our results suggest that PQI differentiated the treatment plans more effectively than the other indices.

The effects of brain irradiation are closely related to the cognitive development of the brain. Merchant et al. reported that each increase in the mean dose of 1 Gy decreases the patient’s IQ by 0.0095, 0.0092, and 0.0088 per month, when treating the total brain, left temporal lobe, and right temporal lobe, respectively [30]. The differences between photon and proton dosimetry in patients with an optic pathway glioma, craniopharyngioma, and medulloblastoma were small but yielded clinically different curves for the cognitive outcomes in a dose–cognitive model [15]. When this model was applied to the results of our study, the IQ deviation between IMRT and PSPT is expected to result in IQ improvements at 70 months after treatment of 1.596, 4.077, and 4.010 at a dose of 30.6 Gy and 3.403, 8.464, and 8.349 at a dose of 55.8 Gy for total brain, left and right temporal lobes, respectively.

## Conclusions

In conclusion, PBT provides superior tumor conformity and normal tissue saving relative to other modalities in the treatment of ICGCTs in various locations. Continued follow-up and systematic assessment of neurocognitive function is necessary for patients undergoing PBT to evaluate whether the extent of normal tissue saving translate into clinical benefits in children with ICGCTs.

## Additional file

Additional file 1:
**Definitions of CI, HI, GI and PQI.**
