# Supplementary material for: Differential dosimetric benefit of proton beam therapy over intensity modulated radiotherapy for a variety of targets in patients with intracranial germ cell tumors
Source: Radiat Oncol. 2015 Jun 26;10:135. doi: 10.1186/s13014-015-0441-5 (PMC4480576; doi:10.1186/s13014-015-0441-5)
Supplement: Additional file 1: — Definitions of CI, HI, GI and PQI. [file 13014_2015_441_MOESM1_ESM.docx]

**SUPPLEMENTARY MATERIALS**

**Definitions of CI, HI, GI and PQI**

[1] Conformity index (CI):

$$CI=\frac{{(PTV volume covered by the prescribed dose)}^{2}}{\left( PTV volume \right)\times(prescribed isodose volume)}$$

[2] Homogeneity index (HI):

$$HI=\frac{D95\%(the minimum doses applied to 95\% of the target volume)}{D5\% (the minimum doses applied to 5\% of the target volume)}$$

[3] Gradient index (GI):

$$GI=\frac{V50\% (\mathrm{the}dose volume receiving more than 50\% the prescribed dose)}{V100\% (the dose volume receiving more than 100\% the prescribed dose)}$$

[4] Plan quality index (PQI):

$$\mathrm{PQI}=\frac{GI}{CI\times HI}$$
